# Evolution of transcription factor function as a mechanism for changing metazoan developmental gene regulatory networks

**DOI:** 10.1186/2041-9139-6-3

**Published:** 2015-01-29

**Authors:** Alys M Cheatle Jarvela, Veronica F Hinman

**Affiliations:** Department of Biological Sciences, Carnegie Mellon University, 4400 5th Ave, Pittsburgh, PA 15213 USA

**Keywords:** Transcription factor, Gene regulatory network, Development, Novelty

## Abstract

The form that an animal takes during development is directed by gene regulatory networks (GRNs). Developmental GRNs interpret maternally deposited molecules and externally supplied signals to direct cell-fate decisions, which ultimately leads to the arrangements of organs and tissues in the organism. Genetically encoded modifications to these networks have generated the wide range of metazoan diversity that exists today. Most studies of GRN evolution focus on changes to *cis*-regulatory DNA, and it was historically theorized that changes to the transcription factors that bind to these *cis*-regulatory modules (CRMs) contribute to this process only rarely. A growing body of evidence suggests that changes to the coding regions of transcription factors play a much larger role in the evolution of developmental gene regulatory networks than originally imagined. Just as *cis*-regulatory changes make use of modular binding site composition and tissue-specific modules to avoid pleiotropy, transcription factor coding regions also predominantly evolve in ways that limit the context of functional effects. Here, we review the recent works that have led to this unexpected change in the field of Evolution and Development (Evo-Devo) and consider the implications these studies have had on our understanding of the evolution of developmental processes.

## Introduction

Gene regulatory networks (GRNs) explain the gene expression states that direct a cell to establish a particular fate [1]. In development, these models describe the mechanisms that take an egg and its localized maternal determinants to an organism with properly placed tissues and fully differentiated cells. GRNs are predominantly composed of intercellular signaling molecules, transcription factor proteins, and *cis*-regulatory module (CRM) DNA, but here we will focus on the transcription factor component. The interaction between a transcription factor and a specific binding site within a CRM allow for positive or negative influence on expression of a target gene. Because these networks instruct the specification of a particular cell type or structure, changes to these networks result in the evolution of animal morphology.

There has been much debate surrounding the mechanisms by which GRNs evolve. Changes to *cis* regulatory modules have historically been considered the dominant source of GRN evolution, and this idea continues to be supported by new data in the genomics era (reviewed in [2–4]). While it is difficult to identify and dissect CRMs and subsequently associate them with a discernible functional divergence, nevertheless, numerous examples have been unearthed (for example, [5–8]). In recent years, genome-wide experiments, such as ChIP-Seq [9] and computational approaches have been instrumental in understanding the contribution of regulatory DNA evolution. For example, using such methods, Schmidt and colleagues detected many instances of lineage-specific gains and losses of binding events, suggesting rapid turnover in *cis* regulatory sequences [10]. Additionally, conserved noncoding sequences, which frequently have regulatory functions, turn over quickly [11].

On the other hand, several lines of evidence suggest that transcription factors are incredibly well conserved over evolutionary time. The first indication of this comes from the now famous examples from the Hox transcription factor cluster. These transcription factors are conserved in both sequence and function, patterning the body axis of organisms as disparate as insects and vertebrates [12, 13]. More recently, this has been shown to extend to cnidarians [14, 15]. These initial discoveries were followed by numerous and particularly compelling functional-equivalence studies in which transcription factors from widely disparate taxa were shown to rescue knock-out phenotypes (for example, [16–18]). In fact, the realization that largely overlapping sets of transcription factors drive the development of essentially all metazoans surveyed lead to the concept of the ‘toolkit for development’ and the birth of Evo-Devo as a discipline [19, 20].

Even prior to this breadth of experimental evidence in support of CRM change as the primary driver of GRN evolution, some theorized that this would be the case [21]. The logic of this argument is as follows: transcription factors are pleiotropic, meaning that they are multifunctional, and thus, mutations that might result in adaptive changes in one context will almost certainly be detrimental to the organism in others. Meanwhile, CRMs are highly modular. A single gene frequently will be regulated by a separate CRM in each of its temporal and spatial expression domains, and therefore one context can easily be altered without affecting the others. Even individual CRMs are modular. CRMs typically contain multiple binding sites for several different transcription factors, each of which can be modified individually. Therefore, it is commonly accepted that transcription factors are under much more constraint than CRMs and, as a result, are less free to evolve changes in sequence and function [4, 22].

More recently, it has been argued that transcription factors also have the capacity to be modular, and consequently could contribute to developmental GRN evolution more significantly than originally considered [23, 24]. These authors maintained that many aspects of protein expression and structure permit protein evolution by reducing pleiotropy. For example, use of tissue-specific splice forms and changes to protein-protein interactions, which will only be relevant in tissues where both interacting proteins are expressed, both offer mechanisms to reduce the pleiotropy associated with transcription factor changes. Recent work has provided even more support for these ideas in addition to revealing unpredicted sources of modularity. Just as genomic approaches have allowed for increased understanding of the contributions of CRM mutations to GRN evolution, bioinformatic, genome-wide, and other novel techniques have also been instrumental to gaining a better insight into the ways in which transcription factors evolve. Here, we survey and synthesize recent experimental findings that support an underappreciated role for transcription factor change in GRN evolution. In particular, we focus on modular protein changes that seem to be favored by evolution, as previously demonstrated by the CRM paradigm, and therefore could occur in other systems. While modular changes will reduce the pleiotropy associated with transcription factor evolution, these changes may still impact the surrounding GRNs in different ways than do CRM changes. Therefore, greater understanding of how both of these GRN components evolve is necessary to understand how species diverge and novel structures are devised.

## Review

### The structure and function of transcription factors are inherently modular

The basic biochemical function of a transcription factor is twofold: (i) to recognize and bind a short, specific piece of DNA within a regulatory region, and (ii) to recruit or bind other proteins relevant to transcriptional regulation, such as other transcription factors, chromatin remodeling proteins, and general RNA polymerase machinery. The first function, DNA binding, directs the transcription factor to its target loci. The second allows the factor to elicit changes in transcriptional levels by influencing the stability of the transcriptional apparatus or the chromatin state. Combined, these functions enable transcription factors to influence gene expression. At the structural level, transcription factor proteins contain discrete domains for exerting these functions, known as DNA-binding domains and protein-protein interaction domains. Some have more than one of each, and others may perform both functions via a single domain. Because transcription factors have such functional units, which may individually acquire mutations and be lost or gained over time, they are modular just as CRMs are and, thus, have opportunities to evolve in ways that minimize pleiotropy.

In general, DNA binding domains are extremely well-conserved, but the rest of the protein readily diverges between homologs. For example, the aforementioned Hox genes were discovered on the basis of highly conserved homeobox DNA-binding domains [12]. Yet, McGinnis and colleagues noted that while there was 75% or better identity within this domain, their Hox paralogs of interest were essentially unalignable outside of this region. This finding has largely held true for other transcription factor families, for instance, basic helix-loop-helix (bHLH) [25], forkhead box (Fox) [26], and Ets [27] families. As we discuss later, this is not to say that DNA binding domains and properties do not evolve. However, it demonstrates that distinct regions of a transcription factor structure and function can be maintained even while the rest of the protein may be changing in ways that might independently impact function.

**Figure 1:**
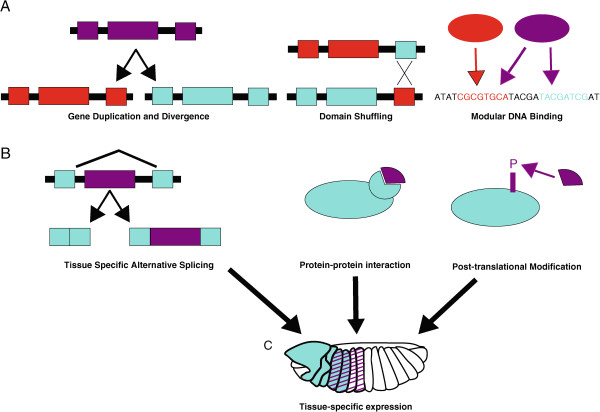
**Mechanisms for generating transcription factor diversity and limiting novel function to specific contexts.** Many of these mechanisms are modular and may be mixed and matched to offer even greater evolutionary flexibility. **A**. Gene duplication, exon shuffling, and modular DNA binding allow transcription factors to increase and change their functionality. While gene duplicates are frequently lost, retention of both copies relaxes constraint and allows the paralogs to diverge through acquisition of mutations (indicated by purple ancestral copy splitting into red and blue versions). Exon shuffling allows transcription factors to evolve new function through acquisition of domains, shown here as a red exon swapped for blue exon. DNA binding can evolve in modular ways too. Here, the red homolog recognizes the red binding site, but the purple homolog can bind both red and blue binding sites. Specificity for the blue site could change without altering functions governed by the red site. **B**. Alternative splicing, protein-protein interactions, and post-translational modifications also increase transcription factor diversity, but these mechanisms also offer context specificity. Alternate splicing can lead to tissues that differ in the version of a transcription factor. Here, the version with the purple exon may have different functional abilities than the all blue version. Protein-protein interactions are particularly important to transcription factor function, since this ability determines whether the protein can successfully alter chromatin or recruit RNA polymerase. However, both interaction partners must be present to exert function, which means that these interactions can be controlled by limiting expression domain **(C)**. Likewise, post-translational modifications are important for altering transcription factor modularity, and are context specific owing to the requirement of co-expression with a modifying enzyme. **C**. *cis*-regulatory module (CRM) level control of gene expression, restricts splice variants, interaction partners, and modifying proteins to distinct spatiotemporal contexts.

Here, we will first discuss the mechanisms that can lead to an increase in transcription factor functional diversity and then consider ways that these functions can also evolve context dependence. Both sets of mechanisms allow transcription factor change to be modular, and importantly, multiple mechanisms can be combined to reduce evolutionary constraint even more. This is depicted in Figure 1, where gene duplication, exon shuffling, and modular DNA binding all offer ways by which a transcription factor can take on new abilities (Figure 1A). Alternative splicing, protein-protein interactions, and post-translational modifications allow transcription factor coding changes to be limited to a particular spatiotemporal context (Figure 1B), especially since these are strongly coupled to the expression specificity provided by CRMs (Figure 1C). Each of these mechanisms will be discussed in this review, with a focus on new experimental findings, especially those that highlight transcription factor modularity.

### The rise and expansion of metazoan transcription factor families

Many transcription factor families arose at the base of the metazoan lineage (reviewed in [28]) and many even predate metazoans [29]. However, these families have each undergone series of duplications and divergence, resulting in numerous homologs, which are an important source of novel material for building GRNs [30–32]. Additionally, an increase in the number and types of transcription factors available may have promoted the evolution of multicellularity; it has been suggested that even more transcription factors were added to the repertoire before the process of embryonic development could evolve [33]. Several important developmental transcription factors are not present in the sponge genome, suggesting that the creation of new transcription factors was critical to the evolution of bilaterians [34].

New transcription factor homologs are created in two ways. When species diverge from a common ancestor, each initially is endowed with the same collection of transcription factors, which generates orthologs. Following the split, each set will acquire mutations. Until a novel regulatory mechanism is devised, the orthologous proteins must execute the same tasks in each species as they did in the common ancestor. This means that orthologous transcription factors are under a great amount of constraint and are therefore thought to remain thoroughly conserved. Conversely, paralogs, transcription factors generated by gene duplication events, are much more free to evolve (Figure 1A) (reviewed in [24]). The new transcription factor, as a duplicate, can have several possible fates. Some duplicates are simply lost. Others take on some of the roles of the original transcription factor, lessening the burden on each copy and giving each copy more flexibility to change. This partitioning of function is known as subfunctionalization [35]. Finally, if one copy maintains all of the ancestral roles of the transcription factor, the other paralog will have essentially no constraint and can neofunctionalize. For example, vertebrate A-Myb and C-Myb are thought to have neofunctionalized after diverging from B-Myb; as a result B-Myb can rescue the single *Drosophila* Myb in functional-equivalence assays, but A and C-Myb cannot [36]. This change occurred because the ancestor of A and C-Myb acquired a new transcriptional activation domain. Additionally, A and C-Myb diverged from each other through subfunctionalization after they were generated by a gene duplication event, which allowed both to be preserved. C-Myb is SUMOylated at two lysines near its C-terminus, which stabilizes the protein and modifies its function *in vivo* [37]. These residues, and therefore this modification, are not conserved in A-Myb [38]. In this way, generation of paralogs results in modularity within a transcription factor family, because each paralog endows the others with greater freedom to change.

An interesting example of duplication and divergence occurs in the vertebrate steroid hormone receptors, a type of nuclear receptor transcription factor. These transcription factors split into two families with different abilities to recognize both hormone ligands and DNA sequences [39]. The extant vertebrate estrogen receptors appear to have maintained the ancestral capacity for both types of recognition, while the other clade of steroid receptors have evolved novel ligand and DNA specificity [40]. Because these new specificities evolved after gene duplication, the ancestral hormone signaling pathway was maintained.

A more extreme example of this is the nematode supplementary nuclear receptor family (supnrs) (reviewed [41]). In *C. elegans* there are 269 supnrs, thought to correspond most closely to vertebrate Hnf4α, although it is difficult to classify them due to highly diversified DNA binding and ligand binding domains [42, 43]. As many supnrs are expressed, and therefore have not devolved into pseudogenes, it is thought that many have neofunctionalized or subfunctionalized. While some supnrs function very much like Hnf4α, others have evolved, potentially via changes to DNA and ligand binding domains, to function more like other metazoan nuclear receptors (reviewed in [41]).

Radiation of the supnrs is not an isolated example. Other transcription factor families also exhibit lineage-specific expansions, and so this is thought to be an important source of gene regulatory change (reviewed in [44]). Zinc finger transcription factor (ZNFs) subfamilies seem to be especially prone to this phenomenon. The zinc-finger associated domain (ZAD) subfamily underwent extensive lineage-specific expansion in the insect lineage, yet there is only one such protein in the vertebrate lineage [45]. Many of these insect-specific ZAD-ZNF transcription factors are associated with developmental processes and have been implicated in the evolution of the meroistic ovary.

Conversely, a different zinc-finger subfamily, the Krüppel-Associated-Box (KRAB-ZNF), radiated dramatically in tetrapod vertebrate lineages, while only one paralog, Prdm9, exists in invertebrates [46]. Many of these KRAB-ZNF proteins are expressed during early development and are crucial for executing epigenetic reprogramming and other early developmental tasks [46–48]. KRAB-ZNFs have been shown to be under positive selection and have acquired amino acid differences between humans and chimpanzees much faster than other genes [49]. Additionally, many KRAB-ZNFs are differentially expressed in the human brain compared to the chimpanzee brain, suggesting a role in the evolutionary divergence of brain development in these species [50]. Thus, expansions within the developmental toolkit are important to the evolution of developmental processes and potentially even the evolution of development as a process after multicellular animals emerged.

### Creating functional diversity among homologs

Another mechanism for increasing modularity within transcription factor repertoires is exon shuffling. Exon shuffling allows for the creation of new genes by piecing together existing functional domains (Figure 1A). This mechanism has been known to create novel genetic toolkit components, and alter all aspects of the functionality of transcription factors. For example, while both the LIM and homeobox domains are ancient and can both be found in a variety of eukaryotes, the combination of two LIM domains and one homeobox to produce Lhx transcription factors is a metazoan innovation [51]. Secondary loss of this homeobox domain gave rise to the Lmo family of proteins, which affect gene expression by binding transcription factors since they cannot bind DNA on their own [51]. Therefore, the constituent parts of Lhx proteins can be gained and lost in a modular manner. Lhx genes have highly conserved roles in neurogenesis, and it has been suggested that they were co-opted into this process from an ancestral role in specifying primitive sensory cells [52]. Thus, the creation of this transcription factor family via domain shuffling was an important step in the evolution of neurogenic GRNs. Likewise, a comprehensive study of domain-shuffling in deuterostomes revealed that a handful of transcription factors in the vertebrate lineage acquired new transactivation domains that may have been important for the evolution of vertebrate-specific features [53]. Tandem duplication of exons can also accomplish this. For example, the DNA-binding abilities of KRAB-ZNFs are thought to be able to diverge by changing the number of zinc-finger domains in the protein [54]. Nowick and colleagues predict these changes will have effects on target genes known to be involved in neurogenesis, muscle, and limb development, all of which differ between humans and other primates. This mechanism also allowed the COE family of transcription factors to diverge through a tandem duplication of part of the helix-loop-helix domain at the base of the vertebrate lineage [55]. It is suggested that this change might allow vertebrate COE orthologs to make a wider variety of heterodimer pairings. Importantly, such rearrangements occur without necessarily altering the existing components, and therefore might take place without disrupting ancestral functions.

### Evolution of DNA-binding specificity

Perhaps the most unexpected source of transcription factor adaptability is modular DNA-binding. This is surprising partly because functional-equivalence studies implied conserved DNA specificity of both orthologous [17, 18, 56] and paralogous transcription factors [57, 58]. Additionally, DNA-binding domains tend to be well-conserved at the sequence level. Instances of complete DNA-binding divergence have been uncovered, but they are quite rare [59–61]. The inability to assay transcription factor binding preferences in a sensitive and high-throughput way was for a long time a roadblock to such studies. PCR-based methods for discovering DNA-binding preference such as SELEX [62] recover only the highest affinity binding sites, and caused the misconception that protein-DNA recognition follows a simple one-to-one code. Only recently, it was realized that protein-DNA interactions are extraordinarily complex (reviewed in [63]). Newer technologies, such as protein-binding microarrays [64, 65], are able to universally assess DNA-binding preference because all binding sites are assayed simultaneously without amplification steps as in SELEX. Because this technique uses purified proteins, it can be known with certainty that observed differences in sequence recognition between homologs are not due to modifications by, or interactions with, other proteins. This technique has therefore been crucial for recent works that have revealed modularity in transcription factor binding.

Initial studies that made use of protein-binding microarrays unearthed a few surprising findings. First, many transcription factors’ binding preferences are best described by multiple position weight matrices rather than the one matrix [66–68]. These are commonly called primary and secondary motifs, where the primary motif is the most preferred. Collapsing these motifs into one position weight matrix obliterates important nucleotide interdependencies. For example, a transcription factor might bind well to motifs starting with AC or TG, but not AG or TC. However, a single position weight matrix depiction could imply that all of these combinations are equally preferred. Additionally, while closely related paralogs share highly similar primary binding sites, they frequently recognize different secondary binding sites (Figure 1A) [66–68]. Importantly, this phenomenon has been demonstrated in a variety of metazoan species (including mice, nematodes, and flies), and applies to many major transcription factor families (including Sox, Fox, ZNF, bHLH, Ets, and Homeodomain) [66–71]; therefore, these studies suggested an important and widespread source of transcription factor modularity that has only just been characterized in greater detail.

A recent study of yeast C2H2 zinc finger paralogs also found modular differences in DNA-binding [72]. These proteins bind DNA using two adjacent zinc finger domains and can be divided into groups, in which a common canonical motif is bound by all members, and subgroups, which share an additional specific motif. Here, it was found that paralogs from the same group are able to adopt different conformations to recognize alternative binding sites; however the mechanism differs between subgroups. For example, one subgroup has evolved changes within both zinc finger domains that permit an alternate docking geometry, while another makes use of an N-terminal region outside the zinc finger domains to stabilize alternative site binding. In all subgroups, both the canonical and alternative sites are bound with high affinity, indicating that recognition of the common canonical motif is not compromised by this plasticity. This is likely critical to maintenance of ancestral functions. Extensive cataloging of the Fox transcription factor family revealed flexibility in binding over evolutionary time too [73]. Some Fox proteins bind canonical primary and secondary motifs, some bind a completely different motif, termed FHL, and others are bispecific and therefore can use the primary, secondary, and FHL motifs. Intriguingly, preference for motifs like FHL and also for dual specificity has arisen multiple times within the Fox family, but never through changes to the DNA-binding helix. Instead, an N-terminal tail that allows for alternative structural configurations appears to be responsible for modular binding changes. These studies describe important new mechanisms that allow paralogous transcription factors to evolve while avoiding pleiotropic effects, in many cases by preserving binding to a canonical motif. This is a highly unexplored mechanism through which gene duplicates can acquire new function.

Orthologous transcription factors are under greater evolutionary constraint; therefore, until recently it was uncertain whether this type of modularity would extend to these genes. In addition to the differences in Fox paralog families described above, Nakagawa and colleagues also observed that different orthologs of yeast Fox3 exhibit substantial DNA binding diversity [73]. Some recognize the canonical primary and secondary motifs, others use the aforementioned FHL motif, and yet another subset recognizes a different variant, termed FVH. Fox3 orthologs that bind the FVH motif also have divergent amino acids in their DNA recognition helix. These observations suggested that orthologs may be able to make use of the same mechanisms as paralogs to diverge in DNA specificity. However these orthologs diverged between single-celled yeast species, and therefore may be under less constraint than the transcription factors used in metazoan development.

Recent work demonstrates that while metazoan developmental transcription factors may not diverge as dramatically as yeast orthologs, they do seem capable of exploiting modular divergence mechanisms used by paralogs [74]. In this study, it was found that orthologs of a t-box transcription factor, Tbr, from a sea urchin (*Strongylocentrotus purpuratus*) and a sea star (*Patiria miniata*) evolved differences in their secondary binding abilities. Interestingly this secondary motif is also different compared to what has been reported for the vertebrate ortholog of Tbr, Eomesodermin [66]. However, all three orthologs recognize the same primary motif despite 800 million years of divergence time [75]. The mechanism that allowed this change to evolve is not yet known, but these orthologs have differences in DNA-contacting amino acids, which might have caused changes in binding specificity. Interestingly, Tbr is known to have different developmental functions in the sea urchin compared to the sea star. In the sea star, Tbr has roles in the development of the endomesoderm and also in the ectoderm [5, 76, 77]. However, in the sea urchin, Tbr’s only function is in skeletogenesis [78, 79]. Changes to Tbr’s DNA binding abilities over the course of echinoderm evolution may be responsible for differences in the developmental roles of this protein.

Several studies have demonstrated that these secondary and other non-canonical alternative binding sites are not only functional *in vivo*, but in many cases have distinct developmental tasks. Notably, in the case of Hedgehog-responsive genes used during *Drosophila* development, low-affinity, non-canonical alternative Cisites cannot be replaced by canonical, higher affinity sites as this results in a switch from activation to repression [80, 81]. As a result, these sites convey important positional information across the anterior-posterior axis during development. In another example, it was found that differences in secondary motif specificity among homeodomain paralogs allows each to execute a particular regulatory program during *Drosophila* muscle development; all have the same primary motif and therefore would not be able to confer different myoblast identities without these unique secondary motifs [69]. Thus, secondary motifs are not an artifact of the protein-binding microarray technology, and exhibit *in vivo* functionality just as primary sites do. Because primary and secondary sites have distinct functions, the effects of changing binding to one type of site are less pleiotropic than changing binding to a solitary binding site. Importantly, since alternative binding sites can be gained and lost without affecting a conserved site [72–74], these developmental functions can be uncoupled and evolve independently, thus relieving constraint on developmental processes and allowing for more diverse cell types and structures to arise.

It has been suggested that use of high affinity primary and lower affinity secondary sites during development could be important to coordinate the timing of different developmental events through a temporal protein gradient [74]. For example, during eye development, proper timing of *Pax6* expression is controlled by the affinity of the Prep1 binding sites within its enhancer [82]. The endogenous sites are low affinity, and replacing these with higher affinity sites causes *Pax6* expression to begin too early. Heterochrony, or shifts in the rate or timing of developmental processes, is an important source of morphological differences between species (reviewed in [83, 84]). Modular evolution of binding site preference and affinity could explain some cases where shifts in relative timing occur, because it allows for coupling and decoupling of processes coordinated by, but differentially responsive to, the same spatiotemporal protein gradient.

### The contextual specificity of transcription factors is inherently modular

In addition to having modular structure and function, transcription factors can also evolve reduced pleiotropy by limiting the spatiotemporal context of their functions. As mentioned previously, temporal control is crucial to the faithful execution of developmental programs, and shifts in timing can alter development and lead to the evolution of morphological changes. Thus limiting a transcription factor’s action to a particular developmental period is important to the process. Likewise, control of the transcription factor’s spatial domain is important for developmental processes. GRNs typically make use of combinatorial logic; thus, addition or subtraction of a constituent transcription factor results in alterations to where the GRN is active. Such changes can modify development and resulting morphology. Therefore, mechanisms are in place to ensure not only that transcription factors are expressed and active in particular developmental contexts, but also to allow the many processes they participate in to be uncoupled and evolve independently. The most well-known, and probably also the most common, mechanism for limiting and altering spatiotemporal contexts is by control of transcription factor expression through CRMs(reviewed in [3, 4]). Here, we highlight ways in which modifications to transcription factor coding regions can allow for context specificity and, thus, also reduce pleiotropy.

### Context-dependent use of domains

Alternative splicing can evolve to produce lineage-specific variants of transcription factors in a modular way from the existing structural composition. This is thought to be particularly useful in the evolution of developmental GRNs because different variants can be limited to a particular tissue or developmental stage (reviewed in [23]). This mechanism is reminiscent of CRM evolution, but offers an opportunity to change the functional ability of the protein through inclusion or exclusion of exons carrying protein-protein interaction or post-translational modification motifs, in addition to limiting the context of isoform expression. Alternative splicing has also been shown to be able to alter DNA-binding domain architecture and, potentially, also DNA-binding specificity in a tissue-specific manner [85]. More recently, Blekhman and colleagues used RNA-Seq to study transcript levels among three primate species and found that the expression of particular splice forms differs between lineages and sexes [86]. In Drosophila, sex-specific abdominal pigmentation patterns require gender-specific splice forms of the transcription factor Dsx, such that the female form activates gene expression and the male form represses expression from the same CRM [87]. Both splice forms use the first three exons of the *Dsx* gene, but the C-terminus of each form is sex-specific due to the retention of exon 4 in the female version, and 5 and 6 in the male version [88]. Interestingly, these splice forms differ in their ability to bind a transcriptional cofactor, Ix [89]. These examples demonstrate that the usage of transcription factor domains is modular and, thus, has the potential to be evolutionarily labile.

### Evolution of protein-protein interactions

Transcription factors do not influence gene expression on their own, but do so as regulatory complexes mediated by interactions between the constituent transcription factors and cofactors. These interactions tend to be context-dependent; a particular protein-protein interaction will only be relevant when both interacting partners are present. The composition of a transcription factor complex is also guided by the types of binding sites present in the CRM, and so many transcription factors participate in multiple non-identical complexes and are able to form interactions with more than one other protein. Therefore, changes to such interactions are predicted to be minimally pleiotropic.

A well-known example critical to arthropod evolution is Ftz, which acquired novel cofactor interactions that changed the function of this transcription factor from homeotic to pair-rule segmentation factor [90]. This occurred through several steps. Change to *Ftz*’s expression domain via CRM evolution was important, but so were changes to the protein coding region. These changes resulted in the loss of an ancestral interaction peptide motif, YPWM, which is required for interaction with Exdand homeotic function, and gain of a new LXXLL motif, which created an interaction with Ftz-F1. The latter confers most segmentation function, although the N terminal arm of the homeodomain also participates. More recently, it was shown that this is not a simple case of drastic changes in a particular lineage. Rather, the YPWM homeotic potential motif evolved into stronger and weaker variants of the ancestral sequence throughout the arthropod clade [91]. While YPWM does not hamper the functionality of the LXXLL motif, it does reduce the residual segmentation ability of Ftz variants that lack LXXLL and, therefore, may impact the evolution of particular Ftz lineages. This suggests an inherent flexibility in this YPWM binding motif that could be co-opted by GRNs to create novelty at other points in the evolutionary trajectory of these organisms. It also suggests that intermediate forms of an adaptive protein change need not be catastrophic to development, which is a common argument against transcription factor evolution as an important component of GRN evolution.

Newly evolved interaction motifs are also able to change the magnitude of an existing function. Throughout bilaterians, the transcription factor Engrailed (En) interacts with a co-repressor Groucho (Gro), usually through a well-conserved motif [92, 93]. However certain groups of insects, namely dipterans and lepidopterans, have an additional, novel Gro interaction motif. This novel motif strengthens the interaction between Gro and En and, as a result, augments En’s existing repressive abilities rather than conferring a novel function on En [94]. An advantage of changing GRNs through CRMs includes the ability to increase or decrease the quantity of a gene product and thus enhance or tone-down its function. This work suggests that the evolution of protein-protein interaction motifs is capable of producing quantitative changes as well.

Importantly, changes to protein-protein interactions can occur without major disruptions of the existing protein-protein interaction domain. Brayer and colleagues discovered that an important new interaction evolved between Hoxa11 and Foxo1a in placental mammals without actually changing the ancestral binding interface [95]. These genes are both crucial to the regulation of gene expression in endometrial stromal cells, and adaptive changes in Hoxa11 had already been shown to be a driving force in evolution of pregnancy in mammals [96]. Without Foxo1a, Hoxa11 represses the expression of pregnancy-related genes instead of activating them, so the advent of the Foxo1a/Hoxa11 interaction is key to the origin of this novelty [97]. Interestingly, the binding interface of these proteins did not change; in fact, Foxo1a had not evolved much at all as evidenced by the fact that eutherian Hoxa11 is able to interact with non-mammalian orthologs of Foxo1a [95]. This is critical because Hoxa11 interacts with Foxo1a via its homeodomain, which is used in other essential functions of this transcription factor such as DNA-binding. The authors suggest that the causative amino-acid changes most likely produced a conformational difference in the protein in the eutherian lineage that makes a pre-existing binding interface accessible to Foxo1a [95].

These case studies highlight the previously underappreciated versatility of transcription factor coding region changes, in addition to offering a mechanism for limiting the context of the evolved transcription factor’s function. Furthermore, they reveal that mutations to transcription factors can accomplish some of the same advantageous functions of CRMs, such as the ability to tweak target gene transcriptional output. Finally, they demonstrate that there are many ways to alter a transcription factor without abolishing ancestral function, such as through changing protein conformation as opposed to the sequence of the functional domain.

### Evolution of post-translational modifications

Post-translational modifications are a common way to increase protein functional diversity. They are of particular interest to those seeking to understand how transcription factors may evolve while avoiding pleiotropy because they are known to regulate the location, longevity, and activity of proteins. They can also allow for alternate protein structure and enhance or prevent protein-protein interactions and DNA-binding (reviewed in [98, 99]). Thus, as is the case for CRMs, the effects of mutations to post-translational modifications can easily be limited to a particular developmental context. Some types of modification, such as phosphorylation, are reversible, and therefore offer even more flexibility.

Moreover, new modification sites evolve rapidly. A comprehensive bioinformatics screen identified over two-hundred ubiquitylation sites that arose in the human lineage since it split from other primates [100]. A similar study also found 37 human-specific phosphorylation sites [101]. Interestingly, it has been suggested that a human-specific protein kinase C phosphorylation site has evolved in the Foxp2 transcription factor, which is important for cortical development and has been implicated in the evolution of speech in humans [102]. It is thought that this modification allowed Foxp2 to enhance its neurogenic function, since the human version has a gain-of-function phenotype in transgenic mice [103].

It is unsurprising then that recent work has found compelling connections between novel post-translational modification sites within transcription factors and the evolution of new features. For example, Ubx, a Hox transcription factor, is expressed in the limb primordia of both insects and crustaceans. Thus, alteration of the Ubx protein explains differences in appendage number between different groups of arthropods rather than CRM level changes [104]. Taghli-Lamallem and colleagues found that an important difference in Ubx among arthropods involves loss of CK2 phosphorylation sites in the insect lineage [105]. Ubx represses the expression of*Dll*, which also results in repression of limb formation. They demonstrated that phosphorylation of CK2 sites in crustaceans interferes with the ability of Ubx to repress *Dll*, and as a result more appendages form in crustaceans compared to insects. The molecular consequence of phosphorylating these sites is unknown, but there are precedents for phosphorylation affecting DNA-binding of Hox proteins and also their protein-protein interactions [106, 107].

Another interesting example entails evolution of pregnancy in mammals, due in part to changes in phosphorylation of Cebpβ [108]. This work demonstrated that a mere three amino-acid changes in an internal regulatory domain, resulting in the loss of two ancestral phosphorylation sites and the gain of a new one elsewhere, completely changed how this transcription factor responds to cAMP signaling. Phosphorylation of the novel site by Gsk-3β is required for Cebpβ to activate the expression of prolactin, an important pregnancy hormone. Developmental GRNs integrate both signaling pathways and transcription factors, and so alteration of the post-translational modifications that connect them offers an attractive way of modifying developmental GRNs.

## Conclusions

Transcription factor coding changes are becoming a theoretically more accepted source of GRN evolution, but there are still only a few studies documenting specific changes and tying those to developmental novelties. Many of the studies we have discussed in this review suggest interesting ways GRN evolution can occur via transcription factor change, but further study is still required in order to understand the full mechanism. As these experimental examples continue to increase, we will be able to decipher what impact these changes have on the wiring of their GRNs and how this might differ from CRM mutations. The original logic supporting CRM mutations over transcription factor changes would suggest that the former are ideally suited to alter the expression of a particular gene and potentially also its downstream targets within a tissue or cell-type, while changes to transcription factors will have broader effects, changing the regulation of large sets of target genes across the organism. The experimental evidence described here points to incremental and modular transcription factor mutations being favored by evolution, and latent motifs and abilities becoming more pronounced or reduced over time. Thus, in many ways, transcription factors evolve in ways that are very reminiscent of CRM evolution in that both use modularity to circumvent pleiotropy. However, it is important to realize that their effects on the surrounding GRNs are potentially not equal. Each type of change may be more ideal for driving different types of GRN changes and developing different types of novelty. More information about both types of change is required to tease out this discrepancy. On the other hand, several recent works suggest that CRM and transcription factor mutations may generally operate together [91, 109, 110]. Additional work will reveal whether such cooperative changes to GRNs are the exception, the rule, or simply another option in creating diverse GRNs, a myriad of developmental processes, and seemingly endless animal forms.

## Abbreviations

bHLH: basic helix-loop-helix
CRM: *cis* regulatory module
Evo-Devo: Evolution and Development
Fox: forkhead box
GRN: gene regulatory network
KRAB: Krüppel-Associated-Box
Supnrs: supplementary nuclear receptors
ZAD: zinc-finger associated domain
ZNF: zinc finger.
